# Housing Risk Factors Associated with Respiratory Disease: A Systematic Review

**DOI:** 10.3390/ijerph18062815

**Published:** 2021-03-10

**Authors:** Nipuni Nilakshini Wimalasena, Alice Chang-Richards, Kevin I-Kai Wang, Kim N. Dirks

**Affiliations:** 1 Department of Civil and Environmental Engineering, Faculty of Engineering, The University of Auckland, 0600 Auckland, New Zealand; yan.chang@auckland.ac.nz (A.C.-R.); k.dirks@auckland.ac.nz (K.N.D.); 2 Department of Electrical, Computer and Software Engineering, Faculty of Engineering, The University of Auckland, 0600 Auckland, New Zealand; kevin.wang@auckland.ac.nz

**Keywords:** housing, health, respiratory disease, risk factors, systematic review

## Abstract

Cold, damp and mouldy housing arises from the degradation of the housing stock over time due to weathering and a lack of maintenance. Living in such houses is associated with many adverse impacts on human health, especially for those with existing health issues. This paper presents a systematic review, using the PRISMA protocol, consisting of an exploratory analysis of housing-related risk factors associated with respiratory disease. The review consisted of 360 studies investigating 19 risk factors associated with respiratory conditions. Each fall into one of four categories, namely, (1) outdoor environment-related factors; (2) indoor air pollution-related factors; (3) housing non-structure-related factors; or (4) housing structure-related factors. The results show that effects of poor housing conditions on occupants’ respiratory health is a growing research field, where poor indoor air quality, mainly due to a lack of adequate ventilation, was found to be the most influential risk factor. Usage of solid fuel and living in an urban area without a pollutant-free air filtration system are the main risk factors related to inadequate ventilation. Therefore, an adequate and reliable ventilation system with air-infiltration was considered to be the main mitigation solution to improve indoor air quality. It is suggested that government organisations and health practitioners could use the identified risk factors to measure the healthiness of existing dwellings and take measures to improve existing conditions and develop regulations for new housing construction to promote the healthy home concept. Further research is needed for risk mitigation strategies to reduce the respiratory health burden attributed to housing.

## 1. Introduction

Housing plays a significant role in the protection of people’s health [1]. Healthy homes with adequate insulation, heating and ventilation, that are pest and contaminant free, and with ample room for those living in the house, have been advocated by the World Health Organization [2]. This is because cold, mouldy, damp or overcrowded houses are known to be linked to adverse health impacts, such as respiratory disease, rheumatic fever and cardiovascular disease [3,4,5,6,7]. In addition, studies have highlighted the association between poor quality housing and the psychological and mental health of residents, in particular, in clinical observation of depression and anxiety [8,9,10].

The world’s population has grown rapidly over the past several decades. According to the United Nations, it will reach 8.5 billion by 2030 and 9.7 billion by 2050 [11]. Thus, governments and decision-makers in many countries across the world are under constant pressure to provide affordable housing to accommodate these growing numbers of people [12]. An additional pressure comes from an aged housing stock. A survey carried out in the USA based on data from 2015 showed that more than 50% of the owner-occupied housing stock was built before 1980, and around 38% of the stock was built before 1970 [13]. Innovations in habitat restoration have been employed in some parts of the USA to repair and mitigate impacts from economy-driven developments and to meet citizens’ needs for improved quality housing [14]. Similarly, in New Zealand, the latest house condition survey showed that around 40% of houses nationwide were constructed before 1966 and are considered to be old and of poor quality [15]. In order to address this issue, between 2016 and 2017, the New Zealand Government spent approximately $285 million to improve the condition of its aging state-owned housing stock. For private residences, however, a problem of rapid ageing and deterioration persists due to low replacement rates and a lack of significant maintenance [16].

Increased acknowledgement of the role that housing plays in health and wellbeing has resulted in some effective solutions emerging from the interface of disciplines of the built environment and public health, with wellbeing- and comfort-centric perspectives adopted recently in various aspects of housing design. However, despite this, housing and climate-related respiratory illnesses persist, and much remains to be learnt.

## 2. Related Works

In 2013, Jackson et al. [17] published a systematic review and meta-analysis examining risk factors for severe acute lower respiratory infection (ALRI) in children under five years. This review included 36 studies investigating 19 risk factors for ALRI, which is the leading cause of child mortality. The review identified low birth weight, lack of exclusive breastfeeding, and crowding as the main three risk factors for ALRI in children. Similarly, in 2015, Shi et al. [18] conducted a systematic review and meta-analysis ascertaining risk factors for the respiratory syncytial virus (RSV) associated ALRI in children under five years. The review consisted of 20 studies investigating 18 risk factors including premature births, low birth weight, being male, having siblings, maternal smoking and a history of atopy. In 2015, Sonego et al. [19] published a systematic review and meta-analysis evaluating risk factors for mortality from ALRI in children under five years. The risk factors associated with socioeconomic status and the environment were young maternal age, low maternal education, low socioeconomic status, exposure to second-hand smoke and indoor air pollution.

Lungs are vulnerable to the outside environment due to the potential for exposure to chemicals, particles and organisms in the outdoor air [20]. As most people in modern society spend about 90% of their day indoors [21], the indoor housing environment also has a significant impact on a resident’s respiratory health. Previous systematic reviews have examined the risk factors associated with respiratory disease in general, including socio–demographic factors, but no studies have involved an in-depth analysis to ascertain the respiratory risk factors associated with poor housing conditions. However, it is vital to investigate all the respiratory risk factors related with poor housing conditions to ascertain the importance of healthy housing in improving an occupant’s respiratory health.

## 3. Aim and Research Questions

This paper analyses and evaluates research articles focused on respiratory risk factors related to housing conditions and indoor environments.

In this paper, we address the following research questions:

1. What are the housing-related risk factors reported in the literature associated with respiratory illnesses and how are they categorised?
2. What are the demographic characteristics of occupants who are susceptible to the identified risks?
3. What mitigation solutions have been implemented to reduce the effects of housing-related risk factors on occupants’ respiratory health?

The following section presents the methodology used for identifying the relevant literature, including the search terms and criteria. The main findings corresponding to each of the research questions are then presented. The applicability of our findings when exploring the role of houses, housing design and maintenance are then presented, as well as possible areas for future research. This review offers clear guidance to the housing and building sectors for identifying key areas for improvement in the design, construction and maintenance of houses. It will also help communities to identify potential threats to a householder’s respiratory health associated with their indoor environment, and to develop personalised preventive measures such as adequate ventilation, the use of clean fuel for cooking and the cessation of smoking inside homes to reduce indoor air pollutant concentrations and thus improve indoor air quality.

## 4. Methodology

A systematic review approach “typically involves a detailed and comprehensive plan, and a search strategy derived a priori, with the goal of reducing bias by identifying, appraising, and synthesising all relevant studies on a particular topic” [22]. It has been widely used in health sector research for many years [23]. However, in recent years, it has also become popular in other domains, such as in engineering [24,25], in the arts and humanities [26,27], as well as in physics and astronomy [28,29]. Compared with a conventional literature review, a systematic review adopts more reproducible, rigorous, explicit and auditable methodologies [30]. In addition, the goal of a systematic review is to provide an answer to a focused question while traditional literature review provides a summary or overview of a topic. The traditional literature review does not follow an established protocol and does not involve a systematic search of the literature, and the process may include an aspect of selection bias [22]. Systematic reviews allow researchers to investigate beyond their immediate area of expertise by following a broad search approach, using pre-defined search terms and uniform inclusion and exclusion criteria [31] to understand the status of the body of knowledge for a particular topic, its trends, as well as revealing theoretical gaps in the existing literature. In addition, a systematic review assesses the quality of the included studies following a set of quality assessment criteria, something not generally considered in a conventional review [31].

**Figure 1:**
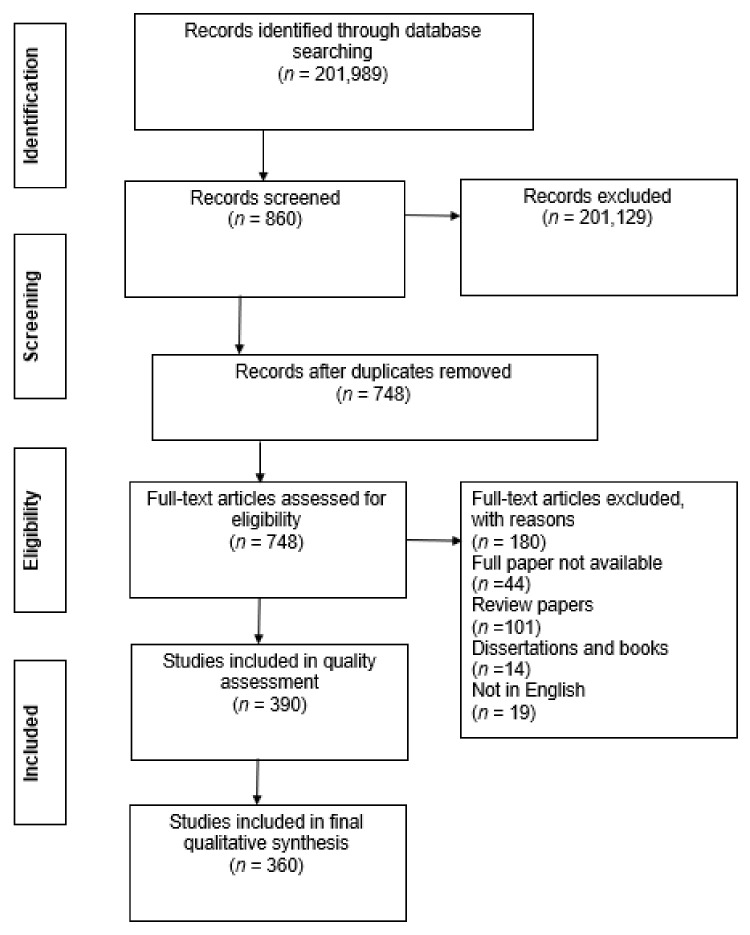
PRISMA protocol of search strategy and results.

A systematic review must follow a guideline to facilitate the reproducibility, transparency and comparability between reviews. The Preferred Reporting Items for Systematic Reviews and Meta-analyses (PRISMA) has been developed to guide systematic reviews and meta-analysis with a flow diagram [23]. It consists of a checklist of 27 items under seven categories, namely, title, abstract, introduction, methods, results, discussion, and funding. The Meta-analysis Of Observational Studies in Epidemiology (MOOSE) contains specifications for reporting meta-analyses of observational studies [32]. This consists of a 35-item checklist under six headings, namely, background, search strategy, methods, results, discussion, and conclusion. This was developed to enhance the usefulness of epidemiological meta-analyses. Since the current study aimed to do a systematic review of respiratory risk factors, the protocol was developed in accordance with the PRISMA guidelines. However, the review process and analysis results reported here are based on a systematic review approach only, without the inclusion of a meta-analysis. Therefore, a correlation analysis between risks was not able to be included. Following the formulation of the research questions, the review was conducted in four steps (see Figure 1). These included: (1) the identification of articles, (2) the screening of relevant papers, (3) the application of eligibility criteria, and (4) the inclusion of articles identified and a synthesis of the findings.

At the identification stage, a search of databases including Scopus, Medline, PubMed, CINAHL, PsycINFO and ERIC was conducted over the period from April 2019 to October 2019. Scopus was selected because it consists of a large number of peer-reviewed journal articles in relevant subject fields, including health science, engineering and social science. Medline contains health science and biomedical information, PubMed focuses on biomedical science, CINAHL covers mainly nursing and allied health areas, and PsycINFO and ERIC are related to complementary medicine and allied health. The search terms used were ‘(respiratory) AND (caus*) OR (factors)’. From the initial database search, 201,989 documents were found.

At the screening stage, the focus was on the relevance of the articles and the removal of duplicates. By reviewing the titles and abstracts of the articles, 2,201,129 articles were excluded as the topic or the focus of the research was not deemed relevant to the respiratory risk factors related to housing conditions. After further removal of duplicates, a total of 748 documents were retained.

The eligibility stage involved a further checking of the relevancy of articles by exercising four inclusion and exclusion criteria. Firstly, it was noted that the number of respiratory deaths in the world has decreased over the past few decades. However, there has been a decrease in the rate of decline since 2006 [33]. Therefore, only publications from 2006 to 2019 were included in the review. Secondly, publications which explored respiratory risk factors related to occupational exposure were excluded as the primary focus of this review is to evaluate housing related risk factors. Thirdly, review and discussion papers were excluded because they contain secondary data only. Books and dissertations were also excluded as these sources are variable in their level of peer review. Lastly, the relevance of each article was assessed according to an evaluation scale that was previously used by Laryea and Ibem [34] and Babalola et al. [35]. The scale was applied based on the extent to which empirical or clinical findings of correlational relationships between a factor and a respiratory health condition of residents was a subject of the article, where “1” denotes low relevance, “2” medium relevance and “3” high relevance. As a result, all articles with content related to real examples, case studies and clinical evidence were rated “3” and were included in the review. Articles rated as “2” or “1” were excluded. The authors independently applied the inclusion and exclusion criteria to the studies analysed. Any discrepancies between the authors were resolved through discussion until consensus was reached. Finally, the eligibility assessment resulted in a total of 390 publications.

**Figure 2:**
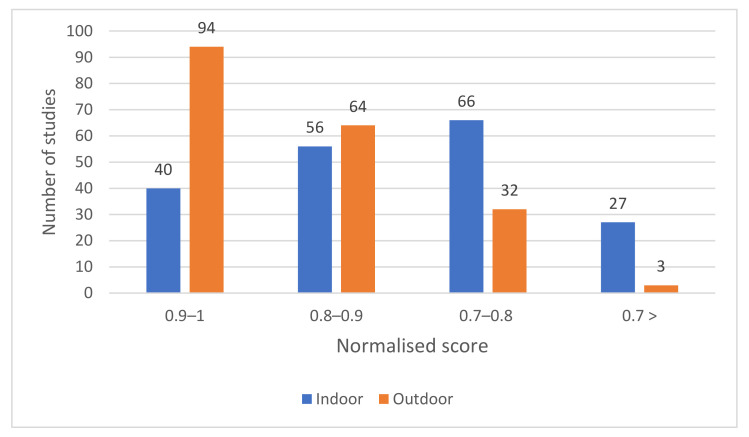
Quality assessment of the studies included in the review.

In the final inclusion stage, a methodological quality assessment of each article was undertaken. The methodological quality of all 390 articles was measured using the Quality Assessment Tool for Observational Cohort and Cross-Sectional Studies [36]. The assessment tool comprises 14 criteria (see Appendix A) to ascertain the reliability of the findings with a score of “1” (Yes) or “0” (No) assigned for each criterion. A summary score for each study was calculated by adding the criterion score and dividing by the total possible score (ranging from 0 to 14). If a criterion did not apply to a specific study design, it was recorded as “not applicable” and was omitted from the summary score calculation. Possible summary scores ranged from 0 to 1, with higher scores representing higher methodological quality. From the 390 studies, five studies employed case studies and three studies employed estimation modelling for which there are not criteria, thus no quality assessment was conducted. Figure 2 demonstrates the summary scores for the 382 articles.

According to the quality assessment, most of the studies had sufficiently specified the research question and the study population (Criterion 1 and 2). In contrast, “sample size justification” (Criterion 5) was the category achieving the lowest average score; only around 9% of the articles justified their sample size. However, for 26% of the studies, the sample size justification was not applicable since these studies analysed the entire population in a particular study area. Despite this, 65% of the studies did not present their reasons for the selection of their sample size, nor provide any indication of the statistical power. It is necessary to have a justification for a chosen sample size when deciding whether or not a study has an adequate number of respondents to be able to identify a genuine correlation, if it exists.

The summary scores of the 382 articles ranged from between 0.46 and 1.00. A summary score of 0.7 was assumed as the threshold value, with articles with a summary score of less than 0.7 excluded from the final analysis, as suggested by Tan and Goonawardene [37]. As a result, a total of 352 articles were found to meet this quality criterion. Including the eight articles that adopted case study methodologies and estimation modelling, a total of 360 articles were included in the final analysis. The information that was extracted from the reviewed articles included the publication source, chronological distribution, geographical distribution, demographic distribution, risk factor categorisation and mitigative solutions. This information was entered into Excel for management and analysis.

## 5. Results

### 5.1. Publication Sources of the Reviewed Articles

Table 1 lists the number of articles (and percentage of the articles included in the systematic review) published in each journal. Peer-reviewed journals represented the highest contribution, with 99% of the selected articles (358 articles), followed by academic conference papers at 1% (2 articles). Among the 147 journals, articles were most frequently published in Science of the Total Environment (18 articles), Environmental Research (18 articles), International Journal of Environmental Research and Public Health (15 articles), Environmental Health Perspectives (15 articles) and Environment International (13 articles). The remaining 67.0% of the journal articles were published across 138 other peer-reviewed journals.

### 5.2. Chronological Distribution of the Reviewed Articles

**Figure 3:**
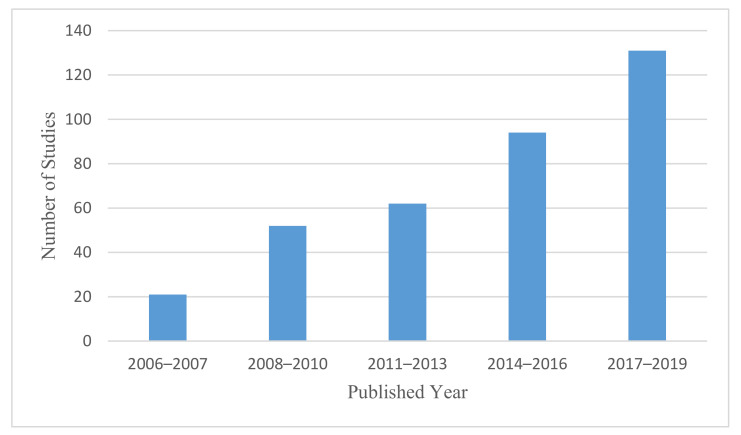
Chronological distribution of reviewed articles.

As shown in Figure 3, over the study period from 2006 to 2019, there was an increasing number of articles published on respiratory diseases and housing-related contributors. While 26% of the studies (94 articles) were carried out in the three-year period between 2014 and 2016, around 36% (131 articles) were conducted in the three-year period between 2017 and 2019.

### 5.3. Geographical Distribution of Identified Articles

**Figure 4:**
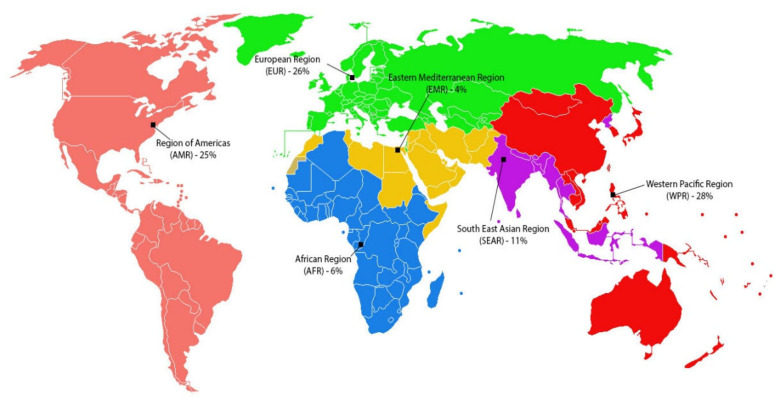
Review articles by WHO regional groups.

The 360 articles were written based on data from 67 countries and from six different WHO regional groups, as shown in Figure 4. Of these, 101 (28%) were conducted in the Western Pacific Region (WPR), 93 (26%) in the European Region (EUR), 90 (25%) in the Americas (AMR), and 40 (11%) in the South-East Asia Region (SEAR). According to the WHO [38], in 2012, at 4066 deaths, the African Region (AFR) had the highest chronic respiratory disease mortality rate per 100,000 people under the age of 70. However, only 6% of the publications identified were conducted in the AFR. It is interesting to note that despite the very low rate of mortality for chronic respiratory disease in China and in the USA (about 1%), 12% and 14% of the articles were based on studies carried out in these countries, respectively.

### 5.4. Demographics of the Population under Investigation

**Figure 5:**
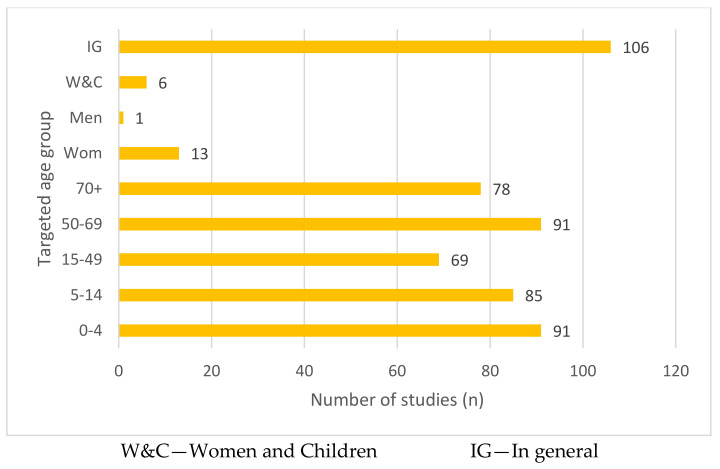
Targeted age group against WHO regional groups.

The demographic information of the population under investigation in the 360 articles is shown in Figure 5 below. Around one-third of the studies (106 articles, 29%) focused on respiratory conditions of people and related risk factors and were analysed based on correlations for the population as a whole, without consideration of demographics. Among the studies that did, greatest attention was given to infants aged between 0 and 4 years old (91 articles, 15%) and children aged between 5 and 14 years (85 articles, 13%). Similarly, elderly people also received attention in the literature, as around 14%, and 12% of the studies in the review ascertained respiratory risk factors to age groups of 50–69 years old and 70+ years old, respectively.

### 5.5. Housing-Related Factors Associated with Respiratory Illness

Analysis of findings in the reviewed articles suggested that housing-related risk factors for respiratory infections can be divided into four categories, namely: (1) outdoor environment factors (194 articles, 54%); (2) indoor air pollution factors (133 articles, 37%); (3) housing non-structure-related factors (18 articles, 5%); and (4) housing structural related factors (15 articles, 4%). Table 2 presents a summary of all of the reviewed articles in relation to risk factors.

**Table 2:** Summary of the reviewed articles.

Factor Ranking	Factor	Category	% of Factor Appearance	Respiratory Diseases (RD)	Pollutant/Effect	Selected Sources
1	Living close to city areas, main roads, highways or industrial areas	Outdoor environment	34%	Acute and chronic respiratory diseases (Asthma, COPD, pneumonia, tuberculosis)	PM, CO, NO_x_, SO_2_, O_3_, VOC	Morgenstern et al. [39], Ji and Zhao [40]
2	Exposure to solid fuel	Indoor air pollution	17%	Asthma, COPD, pneumonia, ARI, URD (both acute and chronic respiratory infection)	PM_2.5_, PM_10_, CO, NO_2_, SO_2_	Da Silva et al. [41], Murray et al. [42], Ranathunga et al. [43]
3	Meteorological factors	Outdoor environment	11%	Asthma, pneumonia, RSV	Extreme temperature and humidity, rainfall and high atmospheric pressure related to an increase in the rate of RDs	Han et al. [44], Fernandez-Raga et al. [45], Son et al. [46]
4	Exposure to ETS	Indoor air pollution	9%	Asthma, ARI, lung cancer, COPD, URD	PM_10_, PM_2.5_, CO, methane	Walker et al. [47], Håberg et al. [48]
5	Moisture or mould damage	Structural related	7%	Asthma, pneumonia, URD	Allergens, bacteria	Park et al. [49], Karvonen et al. [50]
6	Exposure to radon	Indoor air pollution	4%	Lung cancer	Radioactive gas	Bräuner et al. [51], Dempsey et al. [52]
7	Exposure to indoor asbestos fibers	Indoor air pollution	3%	Malignant mesothelioma, lung cancer, lower lung fibrosis	Asbestos fibers	Reid et al. [53], Visonà et al. [54]
8	Carpet floors and HDM	Non-structural related	2%	ARI	Dust, PM	Dales et al. [55], Shendell et al. [56]
9	Presence of pets	Non-structural	2%	Asthma and wheeze	Allergens	Fernandes et al. [57], Dong et al. [58]
10	Exposure to wildland fire	Outdoor environment	2%	Asthma, COPD	PM_10_, PM_2.5_	Liu et al. [59], Shaposhnikov et al. [60]
11	Exposure to VOCs	Indoor air pollution	2%	Asthma, pneumonia	VOCs	Cipolla et al. [61], Jiang et al. [62]
12	Crowding	Non-structural	1%	ARI	Inadequate ventilation	Taksande and Yeole [5], Murray et al. [63]
13	Exposure to dust storms	Outdoor environment	1%	Asthma, ARI	Dust	Trianti et al. [64], Thalib and Al-Taiar [65]
14	Pollen	Outdoor environment	1%	Asthma, eczema, allergic rhinitis	Allergens	Linares et al. [66], Li et al. [67]
15	Use of ozone emitting air cleaners	Indoor air pollution	1%	Asthma, wheeze, dry cough	Ozone	Liu et al. [68], Nguyen et al. [69]
16	Daily cleaning activities (ammonia or chlorine-based cleaners)	Non-structural	0.5%	Asthma	Ammonia	Wang, Su, Hsu, Wang and Wu [4], Medina et al. [70]
17	Drying clothes inside	Non-structural	0.5%	ARI, URD	Facilitate the growth of mould spores and HDM	Mengersen et al. [71], Turunen et al. [72]
18	Living close to mines	Outdoor environment	0.5%	Asthma, lung cancer	Dust	Herrera et al. [73], Pun et al. [74]
19	Exposure to Portland cement dust and volcanic ash	Outdoor environment	0.5%	Lung cancer	Dust	Eom et al. [75], Higuchi et al. [76]

Note: COPD = Chronic obstructive pulmonary disease; ARI = Acute respiratory infection; URD = Upper respiratory disease; RSV = Respiratory syncytial virus; EMR = Eastern Mediterranean region; ETS = Environmental tobacco smoking; HDM = House dust mites; VOC = Volatile organic compounds; PM = Particulate matter; CO = Carbon monoxide; NOx = Nitrogen dioxide; SO2 = Sulfur dioxide; O3 = Ozone.

#### 5.5.1. Outdoor Environment Related Factors

This category includes factors that impact on the respiratory health of people due to outdoor sources, including ambient air pollution and meteorological factors. In the review, the highest number of articles (140 articles) reported the effects of ambient air pollution on human respiratory health. Outdoor air pollution mainly affects residents when they live close to city areas, main roads, highways or industrial areas where fuel combustion pollutants such as particulate matter (PM), CO, NOx, SO_2_ and volatile organic compounds (VOC) present problems. Similarly, occupants living in areas with a high occurrence of wildfires are also likely to suffer from asthma and chronic obstructive pulmonary disease (COPD) due to the extensive inhalation of PM_10_ and PM_2.5_ [77]. People who are exposed to dust storms, pollen, mine and cement dust, and volcanic ash are also vulnerable to respiratory diseases such as asthma, COPD, pneumonia and tuberculosis. The rate penetration of these outdoor pollutants into indoor environments depends on factors such as temperature, the internal volume of the house, window draftiness, size/number/location of windows, and the quality of the air infiltration and ventilation system [78,79]. Therefore, the impact of outdoor sources on the quality of the indoor atmospheric environment can be quite significant when housing conditions are sub-standard.

Meteorological factors also influence asthma, pneumonia and respiratory syncytial viruses (RSV), which were reported upon in 11% of the articles (46 articles) in the review. Air temperature, relative humidity (RH), rainfall and atmospheric pressure are all meteorological risk factors for respiratory disease. Both high and low temperatures are positively related to respiratory illness, but the majority of the research (27 articles) reported on significant correlations with respect wintertime conditions rather than summertime [44,80,81]

#### 5.5.2. Indoor Air Pollution-Related Factors

The factors included in this category are exposure to solid fuel emissions, second-hand smoking and VOCs, which all contribute to poor indoor air quality. Around 17% of the studies in the review detailed adverse effects of solid fuel usage in dwellings on residents’ respiratory health. Kitchens using solid fuel for cooking indicated higher concentrations of PM_10_, PM_2.5_ and CO in comparison with kitchens which used liquefied petroleum gas (LPG) as the cooking fuel source [82]. The pollutant concentration further reduces in households that use electric stovetops for cooking. Around 6% of the studies demonstrated that cooking with LPG and electric stoves are also likely to increase indoor NO_2_ concentrations to which exposure is also related to respiratory disease symptoms. However, air quality is generally better in dwellings that use LPG and electricity as the primary cooking fuel.

Approximately 9% of the publications indicated a positive association between respiratory problems and environmental tobacco smoking (ETS), also known as second-hand smoke (SHS) or passive smoking, the inhalation of the tobacco combustion product of smoking by a non-smoking person [83]. Asthma, acute respiratory infection (ARI), lung cancer and COPD are the most common respiratory conditions associated with exposure to ETS, with the most vulnerable groups being children and adolescents [84]. According to worldwide estimates, up to 40% of children, and 35% and 33% of non-smoking men and women, respectively, are frequently exposed to SHS indoors [85]. If a child is exposed to ETS during their first year of life, there is a higher risk of the development of asthma at 13–14 years of age [86]. It has also been found that pollutants (i.e., PM_10_, PM_2.5_) emitted from smoking react with other compounds and create secondary contaminants (carcinogenic nitrosamines) that increase the risk of respiratory disease in later life [87].

Residential radon exposure and lung cancer incidence were reported by 4% of the studies in the review. Radon is a radioactive natural gas generated by the disintegration of uranium and radium in the earth [88]. According to a study conducted in Spain in 2019, residential radon exposure in lung cancer patients who never smoke was above 200 Bq/m^3^ [89]. Moreover, among the total number of lung cancer mortalities in France in 1999, around 2.2% to 12.4% of deaths attributable to residential radon [88]. Exposure to asbestos fiber is another risk factor for occupants’ respiratory health. Malignant mesothelioma is the main health issue associated with domestic and environmental exposure to asbestos [90,91].

Very few studies (around 2%) examined the associations between respiratory health and exposure to VOCs. Some wooden furniture (mainly chipboard furniture) [92], and some paints and building materials [93] emit high local concentrations of VOCs. However, the rate of emission of VOCs from paint depends on the type and quality of the paint, the nature of the applied surface, and the extent of household ventilation [94]. According to a Russian study, the highest exposures are experienced during and soon after painting, with a lower exposure risk remaining for several months after this [92]. In addition, around 1% of studies analyse the prevalence of respiratory diseases in occupants using ozone emitting air cleaners. Air cleaners purify the indoor air by removing pollutants, but some cleaners also produce ozone [69]. In California, around 10% of households own air cleaners that emit ozone [95]. Children living in houses with personal air purifiers demonstrated a higher prevalence of asthmatic symptoms due to higher ozone exposure in the immediate breathing zone [68].

#### 5.5.3. Non-Structure-Related Factors

This category includes factors that cause unhealthy indoor living environments, either due to the living conditions of households or the living patterns of inhabitants, including carpet floors and house dust mites (HDM), the presence of pets, crowding, daily cleaning activities and drying clothes inside. In comparison with tiled and wooden floors, an increase in dust and viable microorganisms can be found on carpeted floors [96]. Moreover, dust collected from carpeted floors shows a higher HDM allergen concentration. Inadequate maintenance is likely to increase contaminants in carpeted floors and will, therefore, accumulate dust and debris under the carpet. [97]. In general, dust is re-suspended by carpet cleaning activities, and this dust can further increase respiratory disease symptoms such as chest tightness, shortness of breath and morning coughing [71].

The allergens emitted from pets (cats and dogs) also negatively impact human lung function and can lead to exacerbation of asthma and wheeze. According to Behrens et al. [98], exposure to pets during pregnancy and in the first year of life are noteworthy risk factors for asthma and wheeze developing between the ages of six and seven. This shows long-term impacts of early life exposure to pets on the respiratory system.

The negative effects of household crowding on the human respiratory system were discussed in around 1% of the articles. During the rainy season, crowding can become more problematic as people are forced to stay inside [63]. The majority of low- and middle-income families live in overcrowded houses with poor thermal conditions and indoor air quality, resulting from inadequate heating and ventilation systems [99]. Moreover, daily cleaning activities have been shown to be associated with the prevalence of asthma; the chemicals included in cleaning agents have been found to reduce lung function in women who regularly use cleaning products in the home [4].

#### 5.5.4. Housing Structure-Related Factors

The housing structure-related factors category includes factors that result in poor quality indoor living environments due to defects in the building framework, either due to inferior construction methods, workmanship or in the materials used. Household moisture and dampness is the main housing structure-related risk factor for respiratory health, reported in around 7% of the publications included in this review. These studies found positive correlations between mould surface area and respiratory conditions such as asthma, pneumonia and upper respiratory disease (URD). Poor ventilation, cold surfaces, and a relative humidity of greater than 80% are all conditions favourable to mould growth [100]. According to Dales, Ruest, Guay, Marro and David Miller [55], mould surface area was significantly higher in lower-income family homes than in higher-income family homes. This may be due to poorer housing conditions and lower levels of maintenance associated with low-income family houses. The prevalence of dampness also differs according to the occupant tenure status. Becher et al. [101] claimed that higher-income house owners experienced fewer problems of this nature compared with those renting and those economically challenged.

### 5.6. Mitigation Solutions Suggested in the Articles

Table 3 lists the range of mitigation solutions suggested by the publications identified in this review. Since indoor air pollution due to both indoor and outdoor sources is the primary risk factor for respiratory disease, the provision of adequate housing ventilation was considered by a majority of the studies in the review as a viable strategy for improving indoor air quality.

The publications reviewed have suggested improvements in public transport, enhancements in vehicle fleets and fossil fuel substitution to reduce the emission of traffic pollutants as mitigation strategies for reducing outdoor transport-related air pollution levels. Occupants can also install air purifiers in indoor environments to minimise the impact of outdoor air pollution inside dwellings. However, they must be aware to avoid the use of ozone-generating air purifiers as some electronic personal air cleaners emit ozone and can trigger respiratory health issues, such as asthma and wheeze. To reduce exposure to solid fuel emissions, the use of cleaner fuels (LPG or electricity) and the use of cleaner stoves with the provision of adequate ventilation were proposed by 30 and 20 of the publications, respectively.

Dwellings must comply with thermal performance standards (i.e., heating, cooling and insulation) to protect occupants from heatwaves and from cold weather. The installation of an early-warning system to inform occupants of extreme temperature events has been suggested in the literature to reduce the impact of meteorological factors on respiratory health. Furthermore, to minimise the presence of mould and dampness, dwellings should be designed, constructed and maintained appropriately. For instance, landlords and owners alike need to take responsibility for regular maintenance to detect and fix in a timely manner issues related to plumbing and roof leakages, for example. Moreover, residential radon exposure can be minimised by installing a radon-proof membrane or a mechanical barrier across the complete footprint to prevent soil gas entry. In addition, it is vital to measure indoor radon concentrations in high-risk areas and for the government to provide homeowners with incentives or subsidies for remediation works such as the installation of radon sumps or positive pressure systems. Occupants could also significantly improve the quality of their indoor living environment though behaviour change. This includes ceasing smoking inside homes, avoiding exposure to indoor renovation activities, especially amongst vulnerable groups such as expected mothers and infants, avoiding frequent contact with pets, avoiding drying clothes inside and regularly cleaning carpets.

## 6. Discussion

### 6.1. Respiratory Risk Factors Evolving over Time

**Figure 6:**
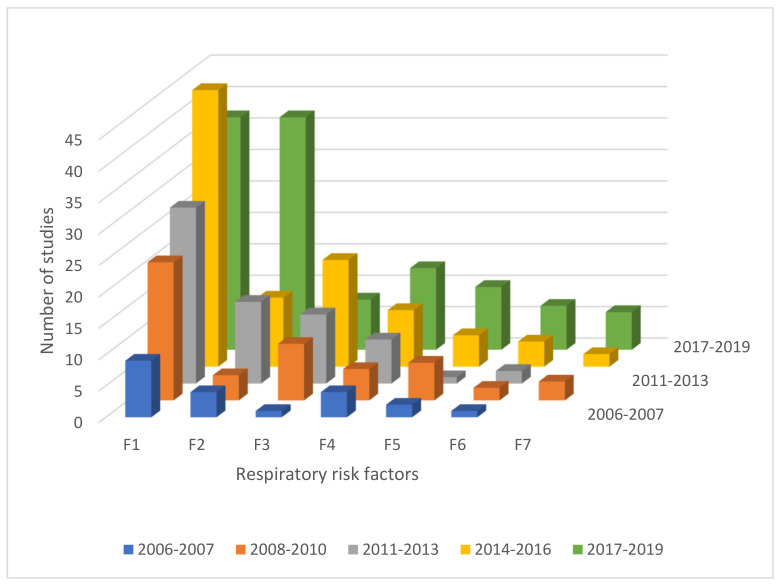
Evolution of consideration of respiratory risk factors in publications over time. Note: F1—Living close to city areas, main roads, highways or industrial areas, F2—Exposure to solid fuel, F3—Meteorological factors, F4—Exposure to ETS, F5—Moisture or mould damage, F6—Exposure to radon, F7—Exposure to indoor asbestos fiber.

A cross-factor comparison of the time evolution of the rate at which respiratory risk factors are mentioned in publications has been carried out. The results of F1–F7 are presented in Figure 6. Since respiratory risk factors F8–F19 were reported by around only 1%–2% of the studies in the review, they were excluded. The results suggest that publications of housing-related respiratory risk factors have increased over the period from 2006 and 2019. There is a tendency for the incrementation in the number of cases along time.

**Figure 7:**
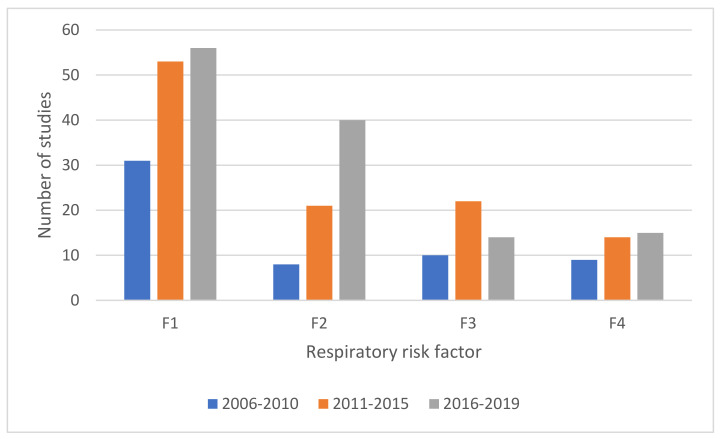
Evolution of most prevalent respiratory risk factors (F1–F4) over time.

As shown in Figure 7, the number of studies considering respiratory health and traffic and industry-related pollutants (outdoor air pollution) has increased over the period from 2006 and 2019. Similarly, Sweileh et al. [102] also found that research on outdoor air pollution and respiratory health has increased noticeably in the period between 2007–2017. According to the WHO, in 2016, 4.2 million people died from exposure to outdoor air pollution [103], a growth from 3.4 million deaths in 1991 [104]. Lelieveld et al. [105] estimated that the premature mortality related to outdoor air pollution could double by 2050. Therefore, the increasing volume of research focused on ambient air pollution and respiratory health impacts depicts the risk of traffic and industry-related pollutants on occupants’ respiratory health.

Moreover, the number of studies related to solid fuel usage and respiratory health grew over the period from 2006 to 2019. The use of solid fuel for cooking and home heating is one of the main primary sources for indoor air pollution which has led to around 3.8 million deaths worldwide annually [106]. In 1980, around 67% (two-thirds) of the world’s population relied on solid fuel for cooking and heating, dropping to about 41% by 2010 (a period of 30 years) [107]. Nevertheless, it remains a significant issue with, according to the World Health Organization [108], around three billion people still relying on solid fuel to fulfil their primary cooking needs.

The number of studies relating second-hand smoking and respiratory health has also increased over the period from 2006 and 2019. Around 600,000 deaths worldwide annually are caused by SHS [109]. Among these, 165,000 deaths are child mortalities, highlighting the significant health burden induced by smokers on non-smokers. According to WHO [109], smokers put around 1.8 billion non-smokers at risk. This might be the reason for increased research over the past several years to examine respiratory health burden from second-hand smoke exposure.

### 6.2. Distribution of Respiratory Risk Factors across Geographical Regions

**Figure 8:**
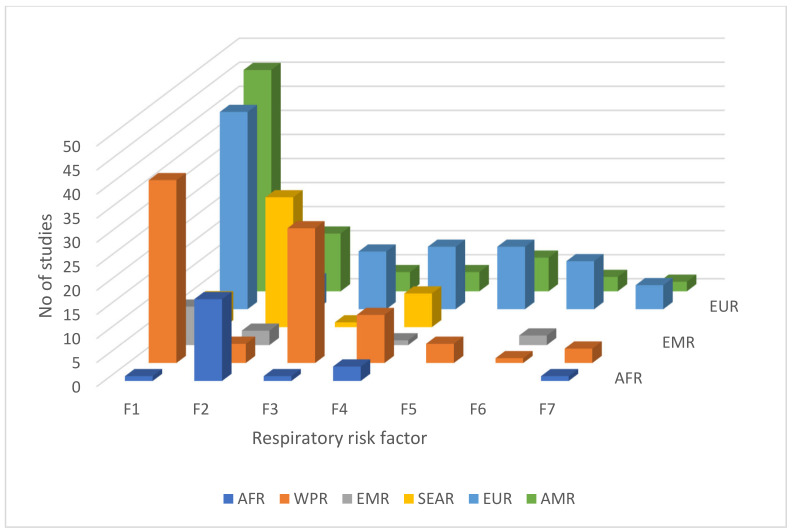
Distribution of respiratory risk factors across geographical regions. Note: AMR—Region of the Americas, EUR—European Region, WPR—Western Pacific Region, EMR—Eastern Mediterranean Region, SEAR—South-East Asia Region, AFR—African Region. F1—Living close to city areas, main roads, highways or industrial areas, F2—Exposure to solid fuel, F3—Meteorological factors, F4—Exposure to ETS, F5—Moisture or mould damage, F6—Exposure to radon, F7—Exposure to indoor asbestos fiber.

Figure 8 illustrates the distribution of studies considering respiratory risk factors undertaken across different geographic regions of the world.

As per the regional comparison, all of the regions studied outdoor-related risk factors more often than indoor, with the exception of those studies carried out in the South-East Asia region (SEAR) and the African region (AFR). In the SEAR, more research was conducted investigating health risks related with solid fuel usage. This is because, according to the WHO [103], the reliance on clean fuels is minimal among SEAR countries as most low and middle-income countries are located in the SEAR and experience the highest levels of household air pollution. Therefore, a large number of studies were carried out to evaluate the effects of solid fuel usage on resident’s respiratory health. In this review, six publications studied both children and women together, and focused on indoor air pollution-related respiratory risk factors. This is because the source of air pollution is mainly biomass fuel usage for cooking, and women and children are the most susceptible groups as they spend more time indoors and near kitchens [110,111].

Low- and middle-income countries experience the highest burden of ambient air pollution with the greatest toll in the SEAR and Western Pacific Region (WPR) [112]. However, only six publications in the review, conducted in SEAR, investigated the impact of traffic-related pollutants on respiratory health, even though the majority of ambient pollution-related deaths over the last two decades were recorded in SEAR [104]. This might be because researchers in SEAR focused more on the respiratory risk factors associated with biomass fuel usage as it has a more direct impact on an occupant’s respiratory health compared with ambient air pollution.

The influence of meteorological factors on occupants’ respiratory health has been predominantly investigated in the WPR (28 studies). Of these, around 50% of the publications (15 in total) were based on studies conducted in China. This could be due to its very high rate of emission of CO_2_ over the past decade (10.15 billion tonnes) [113], and the heightened awareness globally of the impact of meteorological factors on respiratory health [44,114,115,116].

The majority of studies regarding the impact of ETS (or SHS) exposure on residents’ respiratory health have been conducted in the European Region (EUR), WPR and SEAR. This could due to the high exposure rates to SHS in these regions of more than 50% [85]. Most publications that consisted of studies investigating the impact of mould and dampness on residents’ respiratory health were carried out in the EUR, followed by the American Region (AMR) and WPR, in order of decreasing number. No studies were carried out in the SEAR, AFR and EMR during the period of 2006–2019. This may be due to differences in weather patterns and climatic conditions, and the adoption of housing construction materials and methods evident in the EUR, AMR and WPR compared to the SEAR, AFR and Eastern Mediterranean Region (EMR).

The impact of radon exposure on lung cancer has been predominantly examined in the European Region (10 studies). This could be due to the high radon concentrations in the European Region with Sweden, Finland, Czech Republic, Montenegro and Albania being the highest exposed countries in EUR (100–184 Bq/m^3^) [117]. Similarly, the majority of studies regarding the effects of asbestos fiber exposure on occupants’ respiratory health have been conducted in the EUR (5 studies) and WPR (3 studies). According to Delgermaa et al. [118], the highest mesothelioma deaths, which is the main health effect associated of asbestos exposure was found to occur in EUR for the period 1994–2008. This may be the reason why more studies investigating mesothelioma risk associated with occupational and non-occupational asbestos exposure have been conducted in EUR.

### 6.3. Mitigation Measures Associated with the Risk Factors

A range of mitigation solutions has been suggested in different studies. Table 4 illustrates the top nine mitigation measures proposed in the current review. Out of the 360 studies, only 109 (30%) have suggested mitigation actions to reduce risk. The remaining 60% of the articles did not propose any measures highlighting the importance of future research on mitigation solutions to reduce housing-related respiratory health burden.

The use of clean fuels (electricity or LPG) and clean stoves with adequate ventilation for cooking and heating to reduce indoor air pollution was proposed by 28% and 18% of the research, respectively, among the studies that suggested mitigation actions. The use of solid fuel is highest among the SEAR and AFR, with more than 60% of households cooking using solid fuels [107]. The prevalence of solid fuel in other regions ranges from 46% in the WPR, 35% in the EMR and less than 20% in the AMR and EUR [107]. Even though the percentage of households relying on solid fuels for cooking has decreased over the last three decades, with population growth, the absolute number of people using solid fuel has either remained steady globally, or has increased in some regions. Therefore, it is vital to promote the usage of clean fuel and clean stoves for cooking to minimise the health burden attributed to household air pollution.

An improvement in public transport has been proposed as a solution to mitigate ambient air pollution. However, the efficiency of transport systems varies according to different nations. Countries such as Japan, South Korea, Germany and France have advanced and more efficient transportation systems [119] which limit private transportation usage. Therefore, improvement in public transportation is more appropriate in countries with low-income economies such as SEAR countries.

Improvements in indoor ventilation to reduce mould and dampness was suggested in six of the studies in the current review. However, this mitigation strategy is also applicable to minimise indoor air pollution. According to Potts, Rona, Oyarzun, Amigo and Bustos [100], a lack of adequate ventilation is one of the main reasons for increased pollutant concentrations inside houses. However, energy-efficient interventions aimed at improving the airtightness of dwellings entails a decrease in uncontrolled ventilation [120]. Increasing the airtightness of dwellings without the provision of adequate ventilation increase the indoor pollutant concentrations [121]. Since an adequate control of both ventilation and temperature helps to ensure that a dwelling is maintained at a comfortable level of humidity, airtight designs should be combined with an adequate ventilation system and an efficient heat recovery system.

Moreover, an adequate and reliable ventilation system with air-infiltration for removing polluted air from the indoor living environment and replacing it with fresh and clean air is recommended [122]. According to the WHO [103], around 91% of the world population lives in areas where the WHO standards set for outdoor air quality are not met consistently. Also, due to the cold climatic conditions, people are sometimes reluctant to open doors and windows to improve natural ventilation as this would impact significantly on thermal comfort. Therefore, a hybrid or mixed mode of ventilation has been proposed as an intervention for improving building ventilation. Hybrid ventilation systems provide a comfortable indoor living environment by combining both natural and mechanical ventilation systems. This method uses natural driving forces to provide the required airflow, and when the natural airflow is too low, it uses mechanical ventilation [123]. According to Heiselberg [124], properly designed, controlled and installed hybrid ventilation systems can improve household energy efficiency while maintaining adequate indoor air quality and thermal comfort at a lower lifecycle cost.

### 6.4. Challenges and Existing Gaps

Although the literature has suggested mitigation strategies for reducing the impacts of the identified risk factors, the implementation of these solutions can be challenging due to contextual and behavioural issues associated with dwelling occupants. Financial incapability is one of the key challenges, especially those of low and middle income. The challenges related to financial incapability include the usage of solid fuel and inefficient cook stoves, along with the occupancy of dwellings with poor thermal performance. The rate of usage of clean fuel and stoves varies in relation to a country’s economy, with usage ranging from 100% in high-income countries to less than 20% in low- and middle-income countries such as Bangladesh and Malaysia [125]. Since the usage of solid fuel is one of the main sources of indoor air pollution, it is vital to increase the availability of clean fuel for families to reduce the burden of respiratory diseases associated with air pollution exposure. Families of low household income are also at increased risk of experiencing households with poor thermal performance due to inadequate insulation or heating. The increased energy consumption required to heat a poor-performing house further exacerbates the issue.

A lack of education and awareness is another challenge exacerbating the burden of respiratory illnesses. According to Sk et al. [126], children with mothers who are highly educated have around a 50% reduced chance of being affected by respiratory illnesses than those of illiterate mothers. The main issue is an awareness of the adverse impacts of exposure to SHS. Therefore, there is a need for healthcare professionals to help raise awareness about the negative impacts of smoking during pregnancy, as well as the harms of SHS exposure on children and non-smokers, especially in countries where there is a low level of education and literacy [127].

A lack of regulations and standards for reducing indoor generated pollutants is another challenge which increases occupants’ exposure to indoor air pollution. The use of solid fuel is one of the major sources of indoor air pollution, and developing countries mainly rely on solid fuel for cooking and lighting. The existing rules and regulations target the improvement of indoor air quality by promoting the usage of clean fuel and clean stoves with adequate ventilation [108]. However, ventilation standards are mostly made from the perspective of energy consumption. Energy efficiency interventions improve the airtightness of dwellings and thus reduce uncontrolled ventilation [120]. This has the potential to increase indoor generated pollutant concentrations. Therefore, household energy efficiency standards and regulations need to be carefully evaluated to have adequate ventilation to improve indoor air quality. Exposure to SHS is another source of indoor air pollution. In 2006, around 20% of men and 33% of women were exposed to SHS [128]. Even though national-level smoking bans exist in some nations such as Turkey, New York, New Zealand and the United Arab Emirates, around 93% of the global community is left unprotected by 100% smoke-free public policy [129]. Therefore, governments must implement smoke-free policies comprehensively and forcefully to protect their people from SHS exposure.

## 7. Conclusions

The indoor environment has a significant impact on human respiratory health as most people in modern society spend about 90% of their day indoors [21]. Using a systematic review approach, this paper has provided a state-of-the-art analysis of housing-related factors associated with respiratory conditions, with a total of 360 studies published between 2006 and 2019 included in the final analysis. Analysis of the content of these 360 studies revealed that housing-related risk factors that cause respiratory diseases is an evolving research field with the highest interest shown in journal articles. The analysis identified 19 risk factors across four categories: the outdoor environment, the indoor air environment, non-structural housing issues and structural housing issues. Researchers in the South-East Asia Region published most of the selected articles in relation to respiratory health risks caused by solid fuel usage. Scholars in China contributed the highest number of studies about the influence of meteorological factors on occupants’ respiratory health, highlighting the effect of higher CO_2_ emissions on climate change. Concerning the effect of residential radon exposure on respiratory disease, most of the related studies were published in Europe, demonstrating the health burden attributed to higher indoor radon concentrations in European countries.

According to the analysis, a third of selected articles (109 out of 360 articles) proposed mitigation measures for reducing the respiratory health burden associated with substandard housing. Poor indoor air quality, mainly due to a lack of adequate ventilation, was found to be the most influential risk factor for respiratory disease in a household environment. Cooking with solid fuel in poorly ventilated kitchens and living in an urban area without a pollutant-free air filtration system are the main risk factors related to inadequate ventilation. Therefore, the provision of adequate ventilation was considered to be the main mitigation solution for improving indoor air quality.

This paper provides a state-of-the-art systematic review of literature on respiratory disease risk factors associated with housing. The insights derived from this analysis provide a full picture of the categories and types of risks for respiratory health, the geographical distribution of risk categories and mitigation solutions. Such an understanding can be used as a knowledge base for government organisations and health practitioners globally to measure the healthiness of exiting dwellings and take appropriate measures to improve existing conditions as well as develop regulations and standards for new housing. The government can provide fuel allowances or subsidies to promote the use of clean fuel among low-income families and formulate public policies to ensure smoke-free homes to protect children, the elderly and non-smokers from passive smoke exposure. All of these measures can help promote the healthy home concept and reduce respiratory mortalities and morbidities associated with housing. The identification of the geographical distribution of risk factors can be used to take region-differentiated mitigation actions.

All the studies in the analysis examined different housing-related risk factors for respiratory diseases with their effects. However, only a limited number of studies suggested mitigation solutions, highlighting the need for future research investigating mitigation solutions that are “fit for purpose” to reduce the housing-related respiratory health burden. Future research can also look at mechanisms for providing adequate ventilation systems equipped with air filtration to reduce the impact of ambient air pollution, as well as indoor air pollution. This is especially important if the dwelling is occupied by either elderly people or children who are considered at-risk populations.

## Figures and Tables

**Table 1:** Sources distribution of the included articles.

Source	Number of Articles from Source	%	% of Article Type in Selected Literature
**Journals**			
Science of the Total Environment	18	5.0	99
Environmental Research	18	5.0	
International Journal of Environmental Research and Public Health	15	4.2	
Environment Health Perspectives	15	4.2	
Environmental International	13	3.6	
Environmental Health: A Global Access Science Source	11	3.1	
Environmental Pollution	10	2.8	
American Journal of Respiratory and Critical Care Medicine	9	2.5	
PLoS ONE	9	2.5	
Others	240	67.0	
Total number of journals	358		
**Conference papers**			
Conference papers	2	100.0	1

**Table 3:** Suggested mitigation solutions and count of the number of times they are proposed by publications considered in this review.

Respiratory Risk Factor	Mitigation Solutions Suggested in the Literature	Number of Studies in the Review
Living close to city areas, main roads, highways or industrial areas	Improvement in public transport to reduce road traffic	6
	Provide ventilation with pollutant-free air using filtration	5
	Vehicle fleet improvement	5
	Fossil fuel substitution	2
	Close the windows facing roadways	1
	Installation of air purifiers in indoor environments	1
Exposure to solid fuel	Use of cleaner fuel (LPG and electricity)	30
	Use of cleaner stoves with adequate ventilation	20
	Community education on the health effects of smoke from cooking	6
	Have a separate kitchen (indoor or outdoor)	4
	More ventilation in cooking areas	3
	Keep children away during cooking	3
Meteorological factors	Improvement of household thermal performance through heating and insulation	3
	Use air-conditioning during heat waves	3
	Employ an early warning system to inform extreme temperature events	3
Exposure to ETS	Reduce postnatal tobacco smoke exposure by preventing parents from returning to smoking (cessation counselling)	5
	Promote a tobacco-free environment in both households and communities	4
	Increase tobacco taxes and ban tobacco advertising, promotion and sponsorship	4
	Educate families to reduce infant tobacco smoke exposure	3
Moisture or mould damage	Improve indoor ventilation	6
	Effectively repair water leaks	1
	Remove visible mould and dampness	1
Exposure to radon	Installation of radon proof membrane across the complete footprint of the new housing constructions	2
	Use of gas permeable layer, mechanical barrier or gravel foundation to avoid soil gas entry	2
	Installation of an air distribution system to prevent soil air supply	2
	Adequate ventilation and heat recovery system in airtight houses	2
	Installation of radon sumps or positive pressure system	2
	Enhance public awareness on radon exposure risk and methods to reduce radon exposure at homes	5
	Compulsory radon level monitoring and provide incentives or subsidies for remediation works in existing buildings	2
Exposure to indoor asbestos fiber	Installation of dust collectors in an asbestos manufacturing plant to prevent the emission of plant dust to the outdoor environment	1
	Remove asbestos-containing material by trained professional	1
	Restrict or ban asbestos-related products	1
Carpet floors and HDM	Proper maintenance of carpet floors (regular vacuum cleaning)	1
Presence of pets	Reduce the level of endotoxin and dog and cat allergens in homes	2
Exposure to wildland fires	Keep windows closed	2
	Use air cleaners to improve indoor air quality	2
	Employ an early warning system and advise people to stay indoors during wildfires	1
Exposure to VOCs	Adequate ventilation during periods of home renovations	1
	Avoid indoor renovation exposure for expectant mothers and infants	1
Exposure to dust storms	Doors should have proper sealing quality, and houses should be closed during dust storm events	4
	Take measures to reduce wind erosion in desserts	1
	Establish an early warning system for dust storms	1
Pollen	Identify pollen allergen seasons	1
	Replace non-allergic cultivated plant species	1
Use of ozone emitting air cleaners	Have adequate ventilation in rooms with air purifiers	1
	Avoid the use of O_3_ producing air purifiers	1
Daily cleaning activities (ammonia or chlorine-based cleaners)	Produce healthier cleaning products for households	1
	Avoid unnecessary use of cleaning products	1
	Have stricter regulations to reduce chemical exposure. Inform the public by labelling the risks associated with chemicals in consumer products	1

**Table 4:** Top nine mitigation measures proposed in the current review.

Rank	Mitigation Solution	No of Articles	Associated Risk Factor
1	Use of cleaner fuel (LPG and electricity)	30	Exposure to solid fuel
2	Use of cleaner stoves with adequate ventilation	20	Exposure to solid fuel
3	Improvement in public transport to reduce road traffic	6	Living close to city areas, main roads, highways or industrial areas
4	Improve indoor ventilation	6	Moisture or mould damage
5	Community education on the health effects of smoke from cooking	6	Exposure to solid fuel
6	Provide ventilation with pollutant-free air using filtration	5	Living close to city areas, main roads, highways or industrial areas
7	Vehicle fleet improvement	5	Living close to city areas, main roads, highways or industrial areas
8	Reduce postnatal tobacco smoke exposure by preventing parents from returning to smoking (cessation counselling)	5	Exposure to ETS
9	Enhance public awareness on radon exposure risk and methods to reduce radon exposure at homes	5	Exposure to radon

## Data Availability

All data presented in this study are available on request from the corresponding author.

## Appendix A

**Table A1:** Quality Assessment Criteria for Observational Cohort and Cross-Sectional Studies.

1. Was the research question or objective in this paper clearly stated?
2. Was the study population clearly specified and defined?
3. Was the participation rate of eligible persons at least 50%?
4. Were all the subjects selected or recruited from the same or similar populations (including the same time period)? Were inclusion and exclusion criteria for being in the study prespecified and applied uniformly to all participants?
5. Was a sample size justification, power description, or variance and effect estimates provided?
6. For the analyses in this paper, were the exposure(s) of interest measured prior to the outcome(s) being measured?
7. Was the timeframe sufficient so that one could reasonably expect to see an association between exposure and outcome if it existed?
8. For exposures that can vary in amount or level, did the study examine different levels of the exposure as related to the outcome (e.g., categories of exposure, or exposure measured as continuous variable)?
9. Were the exposure measures (independent variables) clearly defined, valid, reliable, and implemented consistently across all study participants?
10. Was the exposure(s) assessed more than once over time?
11. Were the outcome measures (dependent variables) clearly defined, valid, reliable, and implemented consistently across all study participants?
12. Were the outcome assessors blinded to the exposure status of participants?
13. Was loss to follow-up after baseline 20% or less?
14. Were key potential confounding variables measured and adjusted statistically for their impact on the relationship between exposure(s) and outcome(s)?
